# Identification of a Novel Splice Variant Form of the Influenza A Virus M2 Ion Channel with an Antigenically Distinct Ectodomain

**DOI:** 10.1371/journal.ppat.1002998

**Published:** 2012-11-01

**Authors:** Helen M. Wise, Edward C. Hutchinson, Brett W. Jagger, Amanda D. Stuart, Zi H. Kang, Nicole Robb, Louis M. Schwartzman, John C. Kash, Ervin Fodor, Andrew E. Firth, Julia R. Gog, Jeffery K. Taubenberger, Paul Digard

**Affiliations:** 1 Division of Virology, Department of Pathology, University of Cambridge, Cambridge, United Kingdom; 2 The Roslin Institute, University of Edinburgh, Easter Bush, Midlothian, United Kingdom; 3 Viral Pathogenesis and Evolution Section, Laboratory of Infectious Diseases, National Institute of Allergy and Infectious Diseases, National Institutes of Health, Bethesda, Maryland, United States of America; 4 Sir William Dunn School of Pathology, University of Oxford, Oxford, United Kingdom; 5 DAMTP, Centre for Mathematical Sciences, University of Cambridge, Cambridge, United Kingdom; Johns Hopkins University - Bloomberg School of Public Health, United States of America

## Abstract

Segment 7 of influenza A virus produces up to four mRNAs. Unspliced transcripts encode M1, spliced mRNA2 encodes the M2 ion channel, while protein products from spliced mRNAs 3 and 4 have not previously been identified. The M2 protein plays important roles in virus entry and assembly, and is a target for antiviral drugs and vaccination. Surprisingly, M2 is not essential for virus replication in a laboratory setting, although its loss attenuates the virus. To better understand how IAV might replicate without M2, we studied the reversion mechanism of an M2-null virus. Serial passage of a virus lacking the mRNA2 splice donor site identified a single nucleotide pseudoreverting mutation, which restored growth in cell culture and virulence in mice by upregulating mRNA4 synthesis rather than by reinstating mRNA2 production. We show that mRNA4 encodes a novel M2-related protein (designated M42) with an antigenically distinct ectodomain that can functionally replace M2 despite showing clear differences in intracellular localisation, being largely retained in the Golgi compartment. We also show that the expression of two distinct ion channel proteins is not unique to laboratory-adapted viruses but, most notably, was also a feature of the 1983 North American outbreak of H5N2 highly pathogenic avian influenza virus. In identifying a 14th influenza A polypeptide, our data reinforce the unexpectedly high coding capacity of the viral genome and have implications for virus evolution, as well as for understanding the role of M2 in the virus life cycle.

## Author Summary

Influenza A virus is a pathogen capable of infecting a wide range of avian and mammalian hosts, causing seasonal epidemics and pandemics in humans. In recent years, the unexpected coding capacity of the virus has begun to be unravelled, with the identification of three more protein products (PB1-F2, PB1-N40 and PA-X) on top of the 10 viral proteins originally identified 30 years ago. Here, we identify a 14th primary translation product, made from segment 7. Previously established protein products from segment 7 include the matrix (M1) and ion channel (M2) proteins. M2, made from a spliced transcript, has multiple roles in the virus lifecycle including in entry and budding. In a laboratory setting, it is possible to generate M2 deficient viruses, but these are highly attenuated. However, upon serial passage a virus lacking the M2 splice donor site quickly recovered wild type growth properties, without reverting the original mutation. Instead we found a compensatory single nucleotide mutation had upregulated another segment 7 mRNA. This mRNA encoded a novel M2-like protein with a variant extracellular domain, which we called M42. M42 compensated for loss of M2 in tissue culture cells and animals, although it displayed some differences in subcellular localisation. Our study therefore identifies a further novel influenza protein and gives insights into the evolution of the virus.

## Introduction

Influenza A virus (IAV) is a genetically diverse pathogen of global significance, responsible for seasonal epidemics and sporadic pandemics in humans, as well as outbreaks in domestic animals. Its primary reservoir is wild birds, but it can infect a wide range of vertebrate species. For these reasons, there is the need to develop better therapeutics and vaccines [1]. Current vaccines target the surface glycoproteins haemagglutinin (HA) and neuraminidase (NA), but these proteins are subject to antigenic change, necessitating regular updating of the vaccine to ensure a good antigenic match to the circulating strains. Next generation influenza vaccines seek to induce broader or ‘universal’ protection against conserved epitopes; for example, the ‘stalk’ region of HA or the ectodomain of the matrix 2 ion channel protein (M2) [2], [3].

The IAV genome consists of eight segments of negative sense, single stranded RNA (vRNA), each encapsidated into ribonucleoproteins (RNPs) by the viral RNA dependent RNA polymerase and multiple copies of the viral nucleoprotein (NP). Upon infection, incoming RNPs are imported into the nucleus, where the vRNA is transcribed to give positive sense mRNA, and also cRNA, which acts as a replication intermediate. The approximately 13 kb genome has so far been demonstrated to encode up to 13 proteins [4], [5]. Segments 1, 4, 5 and 6 each encode a single protein: PB2, HA, NP and NA respectively. However, segments 2, 3, 7 and 8 have additional protein coding capacity. Segments 2 and 3, whose primary protein products are the polymerase proteins PB1 and PA respectively, additionally produce PB1-F2, PB1-N40 and PA-X proteins from single mRNA species by leaky ribosomal scanning and translation termination-reinitiation in the case of segment 2 and +1 ribosomal frameshifting for segment 3 [4]–[7].

**Figure 1:**
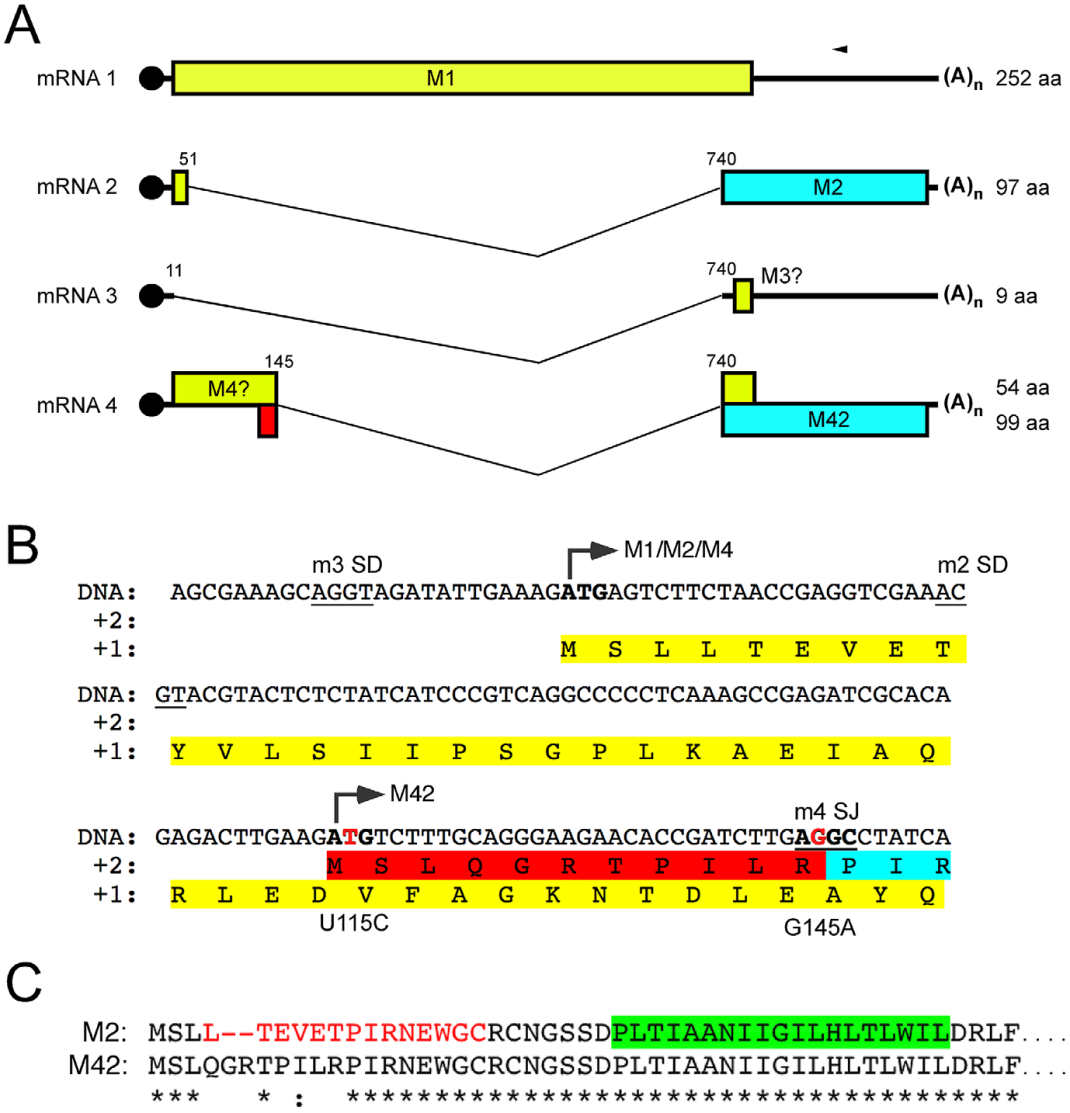
Segment 7 transcript and ORF structure. (A). Diagrammatic summary of mRNA splice variants. The nucleotide coordinates of SD and SA sites are shown. Potential ORFs are colour coded (yellow, M1; blue, M2; red, unique sequence of M42) and total sizes (codons/aa) are given on the right. The arrowhead at top right indicates the binding site of the oligonucleotide used to detect the various mRNA species by radioactive primer extension reactions. (B) Nucleotide sequence (shown as cDNA) and predicted ORFs (colour coded as in (A)) of the 5′-end of PR8 mRNA4. Unused SD sequences and the splice junction (SJ) sequence are underlined. Nucleotides mutated to remove the M42 AUG codon (U115C) or abolish mRNA4 synthesis (G145A) are shown in red. (C) Alignment of the predicted N-terminal sequences of M2 and M42. The range of residues implicated in recognition of M2 by the 14C2 antibody are indicated in red [40]–[42], [61]. The transmembrane domain of M2 is shaded in green.

In segments 7 and 8, protein coding capacity is expanded by differential mRNA splicing. For segment 8, a single spliced species has been described, producing NS2/NEP, while NS1 is produced from the unspliced transcript [8], [9]. Segment 7 mRNA splicing is more complex, as three spliced transcripts have been described (designated mRNAs 2–4) in addition to the unspliced mRNA1 [10]–[12]. Unspliced mRNA1 gives rise to M1 protein. The spliced mRNAs use a common 3′-splice acceptor (SA) site, but use different 5′-splice donor (SD) sites (Fig. 1A). To date, only mRNA2 has been demonstrated to encode a protein: the M2 ion channel [13]. mRNA3 is produced from the most 5′-proximal SD, and is proposed to negatively regulate segment 7 protein expression during early infection [14], a non-essential function for virus growth in tissue culture [15], [16]. More recently, mRNA4 has been shown to be produced by the A/WSN/33 (WSN) strain of virus [12], [15], [17]. It hypothetically encodes an internally deleted form of the M1 protein (“M4”; Fig. 1A) but this protein has not been detected [12].

M2 is a 97 aa integral membrane protein, functional as a homotetramer, with multiple important roles during the virus lifecycle [18], [19]. Each monomer consists of a 24 aa N-terminal ectodomain, a transmembrane α-helix and a ∼50 aa cytoplasmic domain that contains a membrane proximal amphipathic alpha helix [20], [21]. M2 has proton channel activity, which is important for acidification of the interior of the virion upon entry [22]–[24]. In some strains of virus, proton conductance plays an additional role in modulating the pH of the Golgi compartment to prevent premature activation of the HA fusion apparatus [22], [25]. The cytoplasmic tail of M2 also has roles in virus assembly, budding and morphogenesis [26]–[32]. The function of the ectodomain is less well described, although along with the transmembrane domain, it likely plays a role in directing the membrane topology of M2 [33], [34]. It may also be important for incorporation of the protein into virions [35]. Nevertheless, the ectodomain is highly conserved amongst virus strains and this has made it an attractive candidate for a universal influenza vaccine [2], [3].

Surprisingly, it has been possible to generate M2 null viruses, either by introduction of stop codons or by mutating the splice donor site, although these viruses are highly attenuated [15], [36]–[39]. Here, we describe a pseudoreversion mechanism of a virus with a mutated mRNA2 SD site, which reveals a new aspect of IAV biology. After serial passage, we identified a single mutation that upregulated mRNA4 expression without restoring M2 synthesis. Instead, mRNA4 encodes an M2 variant with an alternative ectodomain, designated here M42, which nevertheless functionally complements M2, *in vitro* and *in vivo*. Furthermore, we present evidence that certain strains of IAV, most notably those responsible for the 1983 Pennsylvania outbreak of highly pathogenic avian influenza (HPAI), normally express M42. Our data extend the known IAV proteome and have implications for virus evolution and vaccine design.

## Results

### Pseudoreversion of an M2-null virus

Previously, we used reverse genetics to create an A/PR/8/34 (PR8) virus with synonymous mutations to the mRNA 2 SD sequence. This virus, (M1 V7-T9, hereafter named V7-T9), did not produce detectable levels of M2 and was highly attenuated in tissue culture [37]. To better understand the role of M2 in the virus life cycle, we studied the mechanism by which V7-T9 could regain fitness upon serial passage.

**Figure 2:**
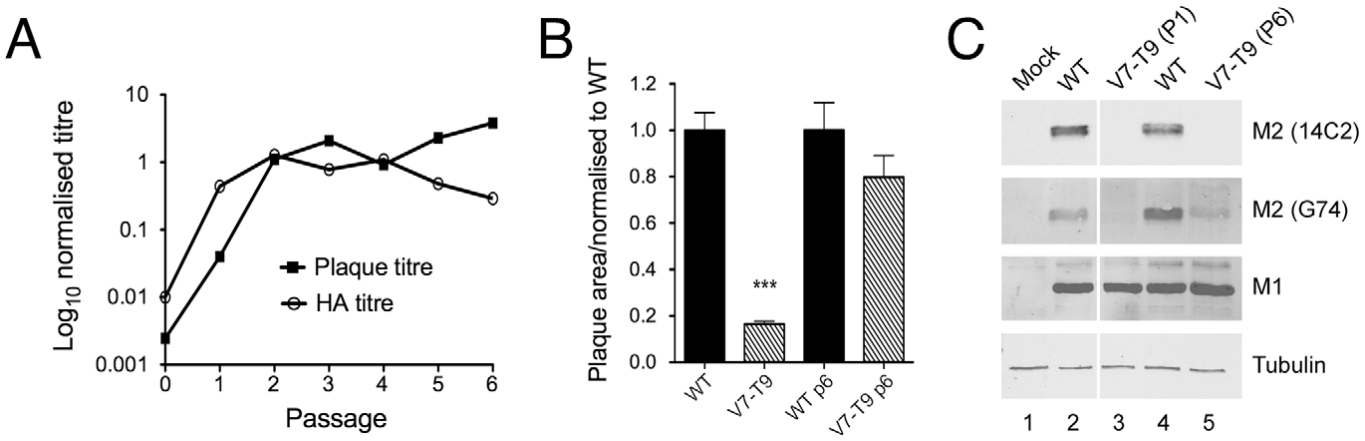
Pseudoreversion of an M2-null virus. (A). Plaque and HA titre plotted before (passage 0) and after each step of 6 serial passage experiments as a fraction of the corresponding mean values obtained from two independent stocks of WT virus passaged in parallel. (B) Average plaque size in MDCK cells of the indicated viruses before and after (P6) serial passage. Values are the mean + SEM of 15–92 plaques normalized to the average WT value from each experiment. *** = p<0.001 compared to WT. (C). Segment 7 polypeptide expression. Lysates from cells infected with the indicated viruses were analysed by SDS-PAGE and western blotting as labeled.

WT and V7-T9 viruses were subjected to six rounds of serial passage via low multiplicity infections of MDCK cells. At each round, outputs were titred by plaque and HA assay. Before serial passage (“P0”), the input V7-T9 virus replicated to a plaque titre 400-fold lower than the WT and had an HA titre 100-fold lower (Fig. 2A). However, on serial passage it regained fitness rapidly, producing similar plaque and HA titres to WT virus within two passages. As a further test of fitness recovery, the plaque areas of the WT and V7-T9 viruses were measured before and after serial passage. Prior to serial passage, V7-T9 displayed a small plaque phenotype ([37]; Fig. 2B). However, after passage six (P6), its average plaque area had increased over four-fold and was no longer significantly different from that of the WT virus (Fig. 2B).

To test if the regained fitness resulted from restoration of M2 expression, we examined infected cell lysates from the original and serially passaged versions of the WT and V7-T9 viruses by western blotting for M1 and M2. All infected cells showed abundant M1 expression, confirming infection (Figs. 2C). Cells infected with the WT virus isolates also contained a polypeptide recognised by the M2 ectodomain-specific 14C2 monoclonal antibody, but as before [37], cells infected with the original V7-T9 virus did not; a phenotype that remained unchanged in the serially passaged isolate (Fig. 2C, lanes 2–5). However, 14C2 antibody recognition is restricted to an epitope encompassing residues 4 to 16 of M2 [40]–[42]. When a polyclonal antibody raised against the entire M2 protein, G74 [43], was used, the original V7-T9 virus still did not show any reactivity (Fig. 2C). However, the P6 V7-T9 virus produced detectable amounts of a G74-reactive polypeptide of similar electrophoretic mobility to that of M2 (Fig. 2C, compare lanes 4 and 5), suggesting that it now expressed some M2 polypeptide, albeit with different antigenicity to the WT protein.

**Figure 3:**
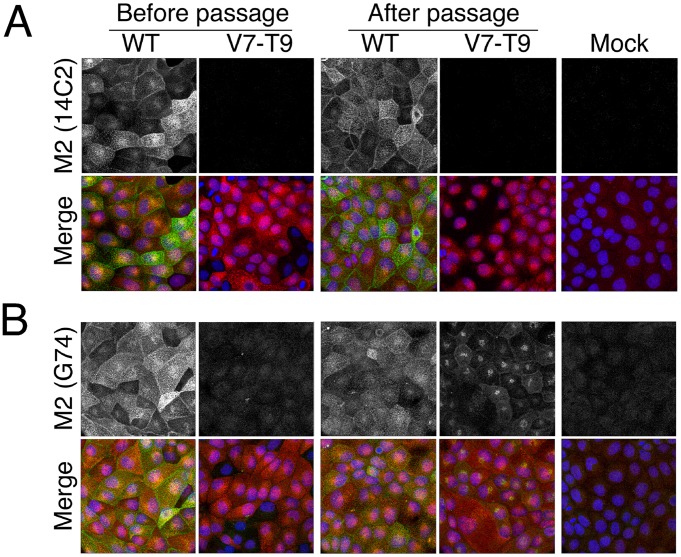
Immunofluorescent analysis of M2 expression. MDCK cells were infected with the indicated viruses at an MOI of 10, fixed at 8h p.i., permeabilised and stained with (A) anti M2 14C2 or (B) G74 (in green, as labeled) and (as counterstains) with anti-NP (red) and DAPI (blue) before imaging by confocal microscopy. Extended focus projections of a series of optical sections through the depth of the cells are shown, either as merged 3-colour images or (in grey scale), the green channel alone.

To further investigate M2 expression by the P6 V7-T9 virus, we examined cells infected with passaged or unpassaged WT and mutant viruses by indirect immunofluorescence for NP (to identify infected cells) and M2, using the two M2-specific sera. All infected cells stained strongly for NP, confirming similar levels of infection (Fig. 3; in red). Consistent with the western blot data, WT virus infected cells also stained strongly with both 14C2 and G74 anti-M2 antibodies, showing the expected predominant staining of apical and lateral membranes [44], while neither isolate of the V7-T9 virus reacted with the 14C2 monoclonal (Fig. 3; in green or separate channel in grey). Also consistent with the western blot data, the unpassaged V7-T9 virus did not stain above background levels with the G74 antiserum, but the P6 isolate showed clear reactivity. However, the staining pattern was markedly different to that shown by WT virus, with prominent perinuclear staining and some staining of lateral membranes (Fig. 3B). Overall, these data suggested that the serially passaged M2 null virus had regained fitness by expressing a variant form of M2 that no longer reacted with the ectodomain-specific antibody.

Following serial passage, segment 7 of both the P6 WT and V7-T9 viruses was sequenced. No changes were detected in the WT virus compared to the reference sequence (GenBank accession EF467824). The P6 V7-T9 virus retained the original mutations that destroyed the mRNA2 SD site, indicating pseudoreversion to recover WT growth properties rather than true reversion. It also contained a single additional change, not seen in the original V7-T9 virus, of a U to A substitution at nucleotide 148 (U148A; mRNA sense, Fig. S1). This change is in the M2 intron and is silent in M1. However, the change would be predicted to improve the mRNA4 SD consensus (Fig. S1), from AG/GUU to AG/GUA [45]. As previously noted, mRNA4 is predicted to encode a 54 aa internally deleted version of M1 [12], from the first AUG on the transcript (Fig. 1B). Notably, the Kozak consensus [46] of AUG1 is not optimal, lacking a G at position 4 (Figs. 1B, S1) and in the context of segment 2, an intermediate strength initiation context AUG1 is known to permit translation initiation at downstream codons by a leaky ribosomal scanning mechanism [4], [6], [7]. Inspection of the segment 7 sequence showed another AUG starting at position 114 in frame 2 (Fig. 1B). The predicted protein product from this AUG would have a variant ectodomain compared to M2, but would be identical from amino acid 10 onwards. The predicted size of the protein product would be 99 amino acids, compared to 97 for M2 (Fig. 1C).

**Figure 4:**
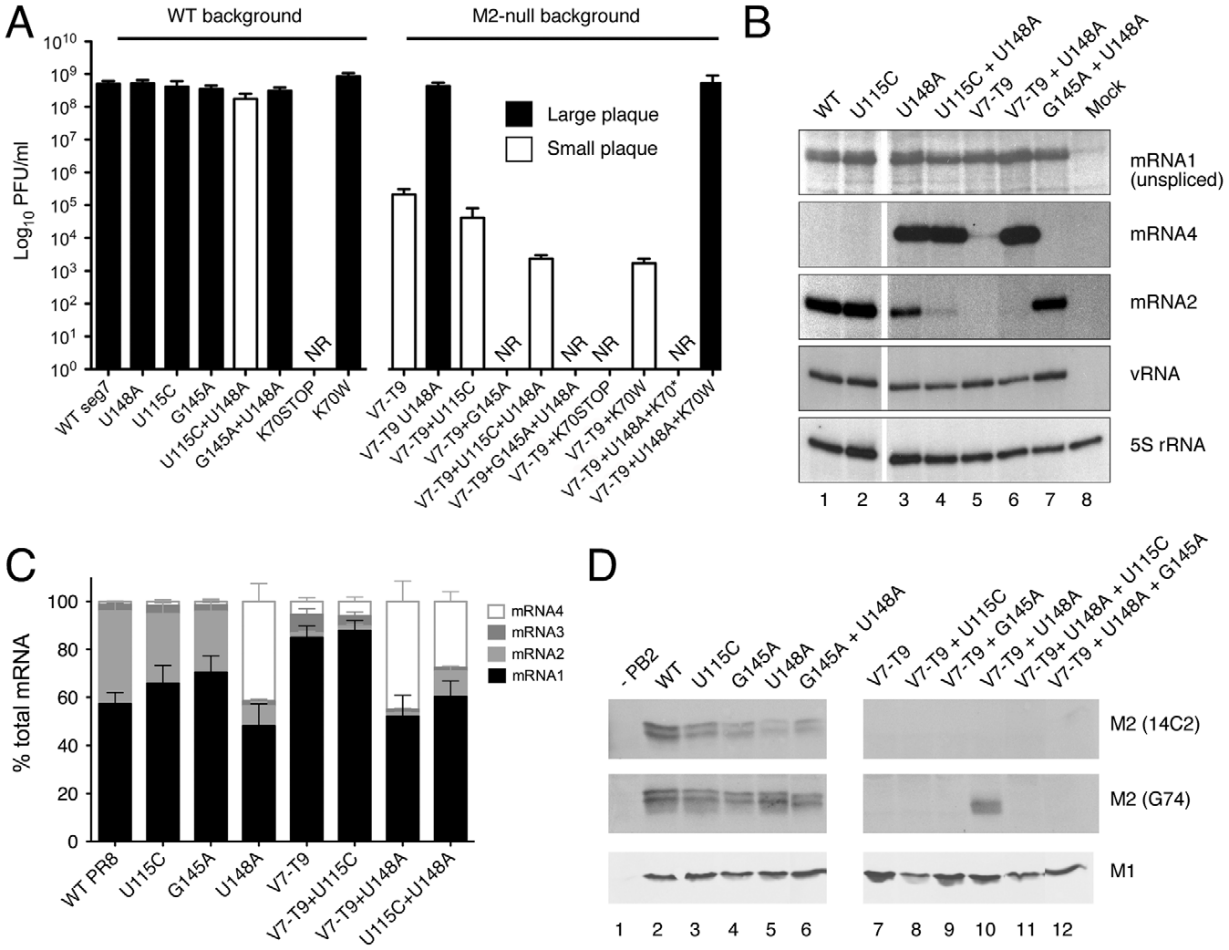
Genetic and biochemical evidence for pseudoreversion through upregulation of mRNA4. (A) Endpoint titres after multicycle replication in MDCK cells of viruses with the indicated mutations to segment 7. Values are plotted are the mean + SEM of between 2 and 18 independent rescues. NR; not rescuable in 2 or more attempts. Viruses were also visually classified into normal (black bar) and small (white bar) plaque phenotypes. (B, C) Segment 7 mRNA accumulation. Total RNA isolated from cells infected with the indicated viruses at 6 h p.i. was analysed by RT-primer extension and urea-PAGE using primers specific for segment 7 mRNAs, vRNA or (as a loading control), cellular 5S rRNA. (C) The amounts of mRNAs 1–4 were quantified by phosphorimager and plotted as the mean ± SD of 3 experiments (D) Segment 7 polypeptide accumulation was monitored by western blot analysis of lysates from 293T cells transfected with reverse genetics plasmids for the indicated viruses at 72 h post transfection with the indicated antisera.

Accordingly, we hypothesized that the U148A change induced pseudoreversion of the V7-T9 virus via upregulation of mRNA4, to produce a variant M2 protein (designated here “M42”) from AUG2 via leaky ribosomal scanning. To test this, we used reverse genetics to first ask whether the U148A change was sufficient to restore WT growth properties to the V7-T9 virus. Initially, a PR8 V7-T9+U148A virus was generated, along with WT, V7-T9 and a virus with only the U148A change. Viruses were rescued by transfecting bidirectional plasmids [47] into 293T cells, amplified by one passage in MDCK cells and plaque titred. WT PR8 grew to approximately 7×10^8^ PFU/ml and formed large plaques, whereas V7-T9 had a small plaque phenotype and was attenuated by approximately 3 log_10_ (Fig. 4A), consistent with previous observations [37]. Introduction of the single U148A mutation into the background of an otherwise WT virus did not alter virus growth properties. However, when the change was added to the V7-T9 background, the double mutant grew to an average of 5×10^8^ PFU/ml and produced normal-sized plaques (Fig. 4A), confirming that the U148A mutation was necessary and sufficient to restore WT growth properties.

To further test the M42 hypothesis, we introduced two mutations that would be expected to block production of the predicted novel polypeptide: either by removing its AUG codon (U115C), or by destroying the mRNA4 SD site (G145A). Each of these mutations was made on the background of WT segment 7, as well as with the V7-T9, U148A or V7-T9+U148A mutations. On a WT background, a virus with only the U115C mutation grew normally and produced plaques indistinguishable from the WT virus (Fig. 4A). When the U115C mutation was combined with the U148A change, the resulting virus grew slightly less well than WT (an average relative titre of 0.44 [n = 4]) and displayed a small plaque phenotype. Addition of U115C to the V7-T9 mutant also had only a minor effect on growth relative to the parent virus. In contrast, its addition to the V7-T9+U148A background reversed the positive effect of the U148A mutation, resulting in a virus that grew poorly (to less than10^4^ PFU/ml) and produced small plaques. Similarly, the G145A mutation had no effect on virus growth as a single mutation or when combined with the U148A change. However, in 3 independent attempts, it was not possible to rescue a virus with V7-T9, U148A and G145A mutations, suggestive of a lethal phenotype.

These data indicated that pseudoreversion of the M2-null virus required mRNA4 and also AUG2. As an additional genetic test of the M42 hypothesis, we introduced a premature stop codon (K70*) into the distal region of the M2 ORF that would be common to both M2 and M42 polypeptides, but outside the M1 (or hypothetical M4) coding region. As a control K70 was also substituted for tryptophan (K70W), a similar sequence change but known to be compatible with M2 function [29]. It was not possible to rescue a virus with V7-T9+U148A+K70stop, although the V7-T9+U148A+K70W mutant grew comparably to WT and V7+U148A viruses. Together, the genetic data are consistent with the hypothesis that the U148A change restores growth of an M2-deficient virus by upregulating expression of mRNA4, allowing expression of an M2 variant from AUG2 of the transcript.

### Identification of M42, a novel M2-related polypeptide

To provide biochemical evidence for the M42 hypothesis, we next examined segment 7 mRNA splicing by the panel of viruses in 293T cells. The V7-T9+U115C+U148A virus grew to insufficient titres to allow high multiplicity synchronous infections and the V7-T9+G145A+U148A virus could not be rescued, so the U115C+U148A and G145A+U148A viruses were used as proxies to analyse the effects of the U115C and G145A mutations on mRNA expression. Following infection, total RNA was extracted and reverse transcriptase-primer extension reactions were performed using a single primer capable of distinguishing segment 7 mRNAs 1–4 [17]. Separate primers specific for segment 7 vRNA and cellular 5S rRNA were also included as controls for virus infection and RNA recovery respectively. The levels of 5S rRNA were equivalent between samples (Fig. 4B), demonstrating equal loading. vRNA levels were also comparable between infected samples, suggesting that all infections had proceeded successfully. The levels of unspliced mRNA1 were also similar between the viruses. However, large differences in the levels of spliced mRNAs 2 and 4 were apparent. In cells infected with the WT virus, the unspliced transcript predominated, but abundant levels of mRNA2 (for M2) were also present (Fig. 4B, lane 1; quantification in Fig. 4C). In contrast, mRNAs 3 and 4 formed minor species that were only visible on long exposure (primary data not shown, but see Fig. 4C for quantification). As expected, mRNA2 was not detected in viruses containing the V7-T9 mutation (Fig. 4B, lanes 5 and 6). Importantly, and as predicted, the U148A mutation, either alone or on a V7-T9 background, strongly upregulated production of mRNA4 (compare lanes 1, 3 and 6). This effect was blocked when the mRNA4 SD was destroyed with a G145A change (lane 7). Interestingly, the changes in levels of mRNAs 2 and 4 were partly reciprocal. Loss of the mRNA2 SD site in the V7-T9 virus was associated with weak upregulation of mRNA4 (compare lanes 1 and 5), while improvement of the mRNA4 SD by the U148A change in an otherwise WT background led to around a three-fold drop in mRNA2 levels (compare lanes 1 and 3). Addition of the U115C change to the U148A virus caused a further decrease in mRNA2 levels, but left mRNA4 levels unaltered (compare lanes 3 and 4). Overall, these data supported the proposed mechanism of pseudoreversion involving increased production of mRNA4.

Next, we analysed segment 7 protein expression from the mutant viruses by western blotting for M1, and for M2 using 14C2 and G74 antisera. Lysates from the primary reverse genetics transfections in 293T cells were used, because V7-T9+G145A or V7-T9+G145A+U148A viruses could not be obtained. Virus polypeptides in these lysates will therefore come from several sources: from RNA Pol II transcription of the bidirectional plasmid, from viral transcription in cells where active RNPs have been reconstituted by transfection, and from spread of viable virus through the cell culture. To control for purely plasmid-mediated expression, lysates from a transfection where PB2 was omitted were probed. In this sample, levels of M1 and M2 were below the limit of detection, although they were readily visualised when all eight plasmids were transfected (Fig. 4D, compare lanes 1 and 2). This suggested that under these conditions, the major signal came from viral gene expression. When mutant and WT transfections were compared, M1 levels were broadly similar between samples, but there was more variation in M2 levels. As expected, 14C2 reactivity was only detected from viruses with an intact mRNA2 SD (lanes 2–6) and was absent from all of the V7-T9 family of viruses (lanes 7–12). G74 reactivity was also readily detectable in all samples from viruses able to make mRNA2. However, in the absence of mRNA2, it was only detectable in the V7-T9+U148A transfected lysates (lane 10). Significantly, this was dependent on the presence of both elevated mRNA4 levels and segment 7 AUG2, as addition of either or the G145A or U115C mutations ablated its expression (compare lanes 10, 11 and 12).

**Figure 5:**
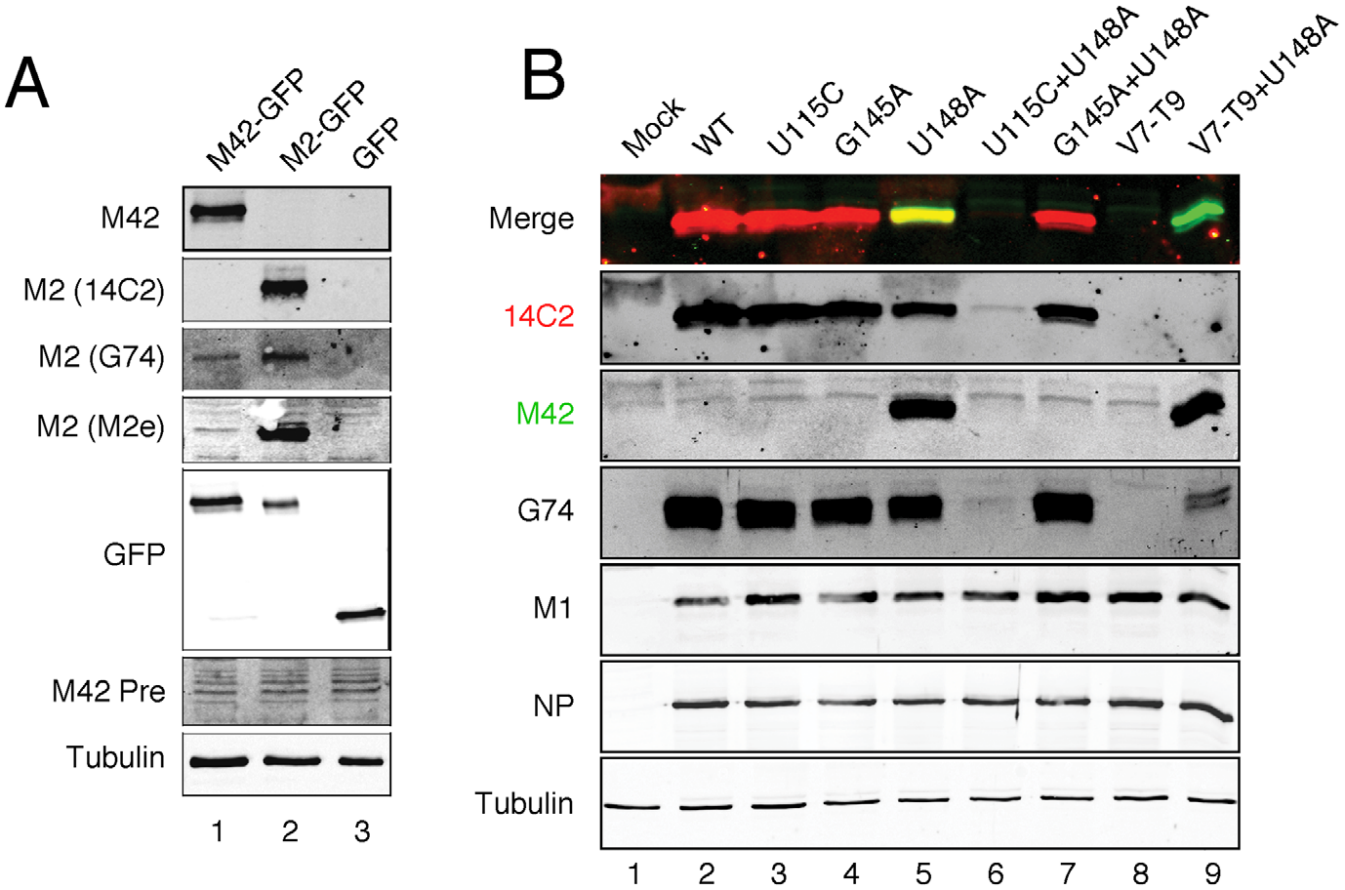
Direct detection of the M42 polypeptide. (A) Validation of anti-M42 serum. Lysates from cells transfected with the indicated GFP polypeptides were analysed by SDS-PAGE and western blotting as labeled. (B) Detection of M42 from virus-infected cells. Lysates from cells infected with the indicated viruses at 10 h p.i. were analysed by SDS-PAGE and western blotting as labeled. The same membrane was probed with mouse anti-M2 14C2 and rabbit anti-M42 using different colour secondary antisera; individual grey scale and colour merged images are shown.

Next, to prove the existence of the M42 polypeptide, we raised a specific antibody against a peptide corresponding to the predicted novel ectodomain of PR8 M42. To validate the serum, we tested it against transfected M42 and M2, both fused to GFP. M42-GFP, M2-GFP or GFP alone were transfected into 293T cells and the resulting cell lysates were probed with anti-M42 and anti-M2 14C2 or G74. Samples were also probed with anti-GFP and tubulin antisera, to confirm expression of the GFP polypeptides and equal sample loading respectively (Fig. 5A). The anti-M42 serum detected M42-GFP with a high degree of specificity over M2-GFP (compare lanes 1 and 2). Conversely, the 14C2 antibody was specific for M2-GFP, while as expected, anti-G74 detected both M42 and M2-GFP. A preimmune bleed from the rabbits immunized with the M42 peptide did not react with either M42-or M2 GFP. To further probe the immunological cross-reactivity between M2 and M42, we tested a polyclonal antiserum raised against the entire M2 ectodomain, anti-M2e [48]. This reacted strongly with M2-GFP and only weakly with M42-GFP, confirming the novel antigenicity of the M42 ectodomain.

Having generated a specific M42 antibody, we investigated expression of the protein in cells infected with the panel of WT and mutant viruses. Western blot analysis of MDCK cell lysates using anti-tubulin sera demonstrated equivalent loading of all samples while anti-M1 and anti-NP sera showed equal levels of infection except in mock infected cells (Fig. 5B). WT, U115C, G145A and G145A+U148A viruses expressed abundant quantities of a polypeptide that reacted with 14C2 and G74 anti-M2 sera (Fig. 5B, lanes 2–5 and 7). In contrast, the V7-T9 virus did not produce detectable levels of any M2-related polypeptide (lane 8). However, concomitant upregulation of mRNA4 on this background by the addition of the U148A mutation led to abundant synthesis of an anti-M42 reactive polypeptide (lane 9), confirming our hypothesis of a novel M2-related polypeptide. The U148A mutation also led to synthesis of readily detectable amounts of the M42 polypeptide when introduced into an otherwise WT background (lane 5). M42 reactivity was however lost on this background by mutation of AUG2 with the U115C change, or by mutation of the mRNA4 SD using G145A (lanes 6 and 7). Consistent with the mRNA abundance data, overall amounts of M2 were reduced by the U148A mutation, as judged by 14C2 and G74 staining (compare lanes 1 and 5). In addition, double staining the same blot with the mouse 14C2 antibody in red and the rabbit M42 antibody in green allowed the creation of a merged image (Fig. 5B, top panel) that illustrates the similar molecular weights of the M2 and M42 polypeptides, as well as their changing relative abundance in response to mutations to SD sites of mRNAs 2 and 4.

**Figure 6:**
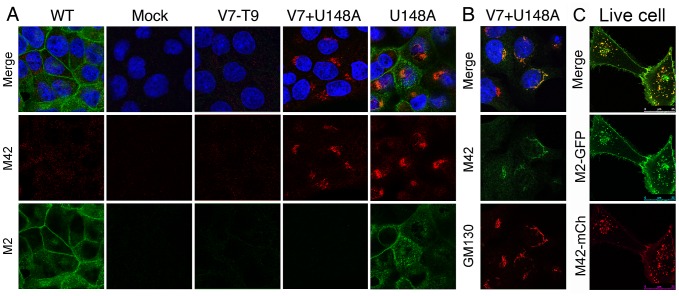
Intracellular localization of M42. (A,B) MDCK cells were infected with the indicated viruses at an MOI of 3, fixed and permeabilised at 10 h p.i. and stained with DAPI (blue), anti-M42 and (A) anti-M2 14C2 or (B) anti-GM130. Single optical sections are shown, either as single channels or merged overlays as labeled. (C) A549 cells were transfected with M42-mCherry and M2-GFP plasmids and imaged at 37°C without fixation 16 h later. Single optical slices are shown. See also corresponding videos S1, S2, S3.

Immunofluorescent staining of P6 V7-T9-infected cells suggested that the two forms of M2 localised differently within infected cells (Fig. 3B). To test if this truly reflected a difference in behaviour of M42 compared to M2, we infected cells with the relevant recombinant viruses and examined M2 and M42 localisation by immunofluorescence. In WT virus infected cells, M2 protein localized to the plasma membrane (visible as staining of lateral membranes in single optical slices through the midline of the cells) as well as internally, often in a perinuclear position (Fig. 6A). Double staining for M42 however, only produced background levels of fluorescent signal, similar to mock infected or V7-T9 infected cells. In contrast, cells infected with the V7-T9+U148A virus did not stain for M2 but stained strongly with anti-M42, with the M42 signal largely present in a perinuclear structure. Confirming that the two proteins did indeed localize differently in infected cells, when cells infected with the U148A virus (which expresses both proteins; Fig. 5B) were examined, the two polypeptides displayed limited colocalisation in the perinuclear region, but were largely in separate regions of the cell (Fig. 6A). Double staining of cells infected with the V7-T9+U184A virus with anti-M42 sera and a variety of markers for the cellular exocytic pathway showed good co-localisation with GM130 (Fig. 6B and data not shown), indicating that M42 was largely resident in the *cis*-Golgi apparatus. To examine intracellular trafficking of M42 further, we created a plasmid encoding an M42-red fluorescent protein fusion (M42-mCherry) and examined the localization of the chimaeric protein in living cells, in comparison to a simultaneously transfected M2-GFP fusion. Both polypeptides co-localised in discrete cytoplasmic puncta and at the plasma membrane, but while the intensity of GFP and mCherry fluorescence was similar in the cytoplasmic dots, there was clearly less of the M42-mCherry protein on the plasma membrane at steady state (Fig. 6C). Examination of time lapse films showed that the cytoplasmic puncta showed the expected pattern of movement for intracellular vesicles (Videos S1, S2, S3). Overall therefore, we conclude that like M2, M42 enters the exocytic pathway, but its altered ectodomain affects intracellular trafficking of the protein, resulting in a lower proportion resident at the plasma membrane.

**Figure 7:**
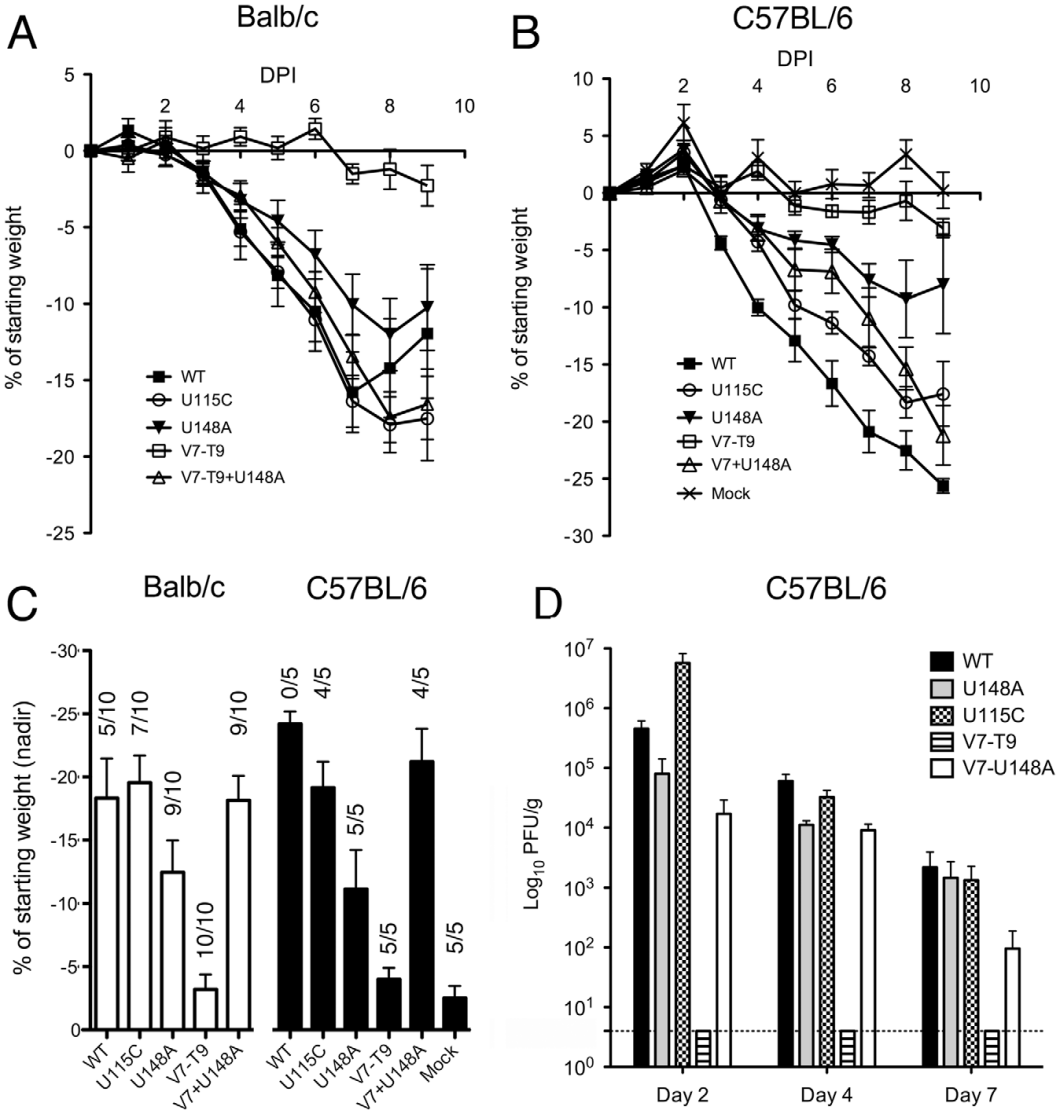
Pathogenicity of segment 7 mutant viruses in mice. Mice were inoculated with 100 PFU of the indicated viruses and (A, B) weight loss measured on a daily basis. Mice were euthanised if their body weights fell below 25% of the starting value. (A) BALB/c mice were used. Data plotted are the mean and SEM values from two independent experiments each using 5 mice/group. (B, D) C57BL/6 mice were used. Data plotted are the mean and SEM values from a single experiment with 5 mice/group. (C) Mean and SEM nadir weights from (A, B) are plotted. Numbers over the bars indicate the number of mice that survived/total group size. (D) Lung titres (mean and SEM) from mice sacrificed at the indicated days. Three mice on days 2 and 7 and four mice on day 4 postinfection were used. Dashed line indicates the lower limit of detection.

The marked difference in intracellular localization between M2 and M42 was surprising, given that the two proteins were apparently interchangeable with respect to virus replication in MDCK cells (Fig. 4A). We therefore tested the effect of modulating M42 expression on virus pathogenicity, using the murine infection model. When either BALB/c or C57BL/6 strain mice were infected with 100 PFU of WT PR8 virus, they lost weight rapidly (Figs. 7A, B), showing average peak weight losses of around 20% and substantial amounts of mortality (Fig. 7C). High titres of virus were also recoverable from the lungs of infected C57BL/6 mice at days 2 and 4 p.i., dropping somewhat at day 7 (Fig. 7D). In contrast, mice infected with the same titer of the M2-null V7-T9 virus showed minimal weight loss, few signs of illness or virus replication and no mortality. However, upregulation of mRNA4 synthesis via U148A on the V7-T9 background substantially increased virus replication and pathogenicity in terms of virus titres andweight loss, although the overall mortality was less than observed with WT virus. Conversely, increasing M42 expression by adding the U148A change on the background of a WT virus still able to express M2 had the opposite effect, decreasing the severity of weight loss and overall mortality, although lung titres were not affected. Removal of the M42 AUG codon with the U115C mutation had little effect in BALB/c mice but led to slightly delayed weight loss and decreased mortality in C57BL/6 mice. Overall therefore, altering the balance between M2 and M42 expression modulated virus pathogenicity, but a virus that only expressed M42 still caused significant disease.

### Production of mRNA4 and M42 in other strains of influenza A

**Figure 8:**
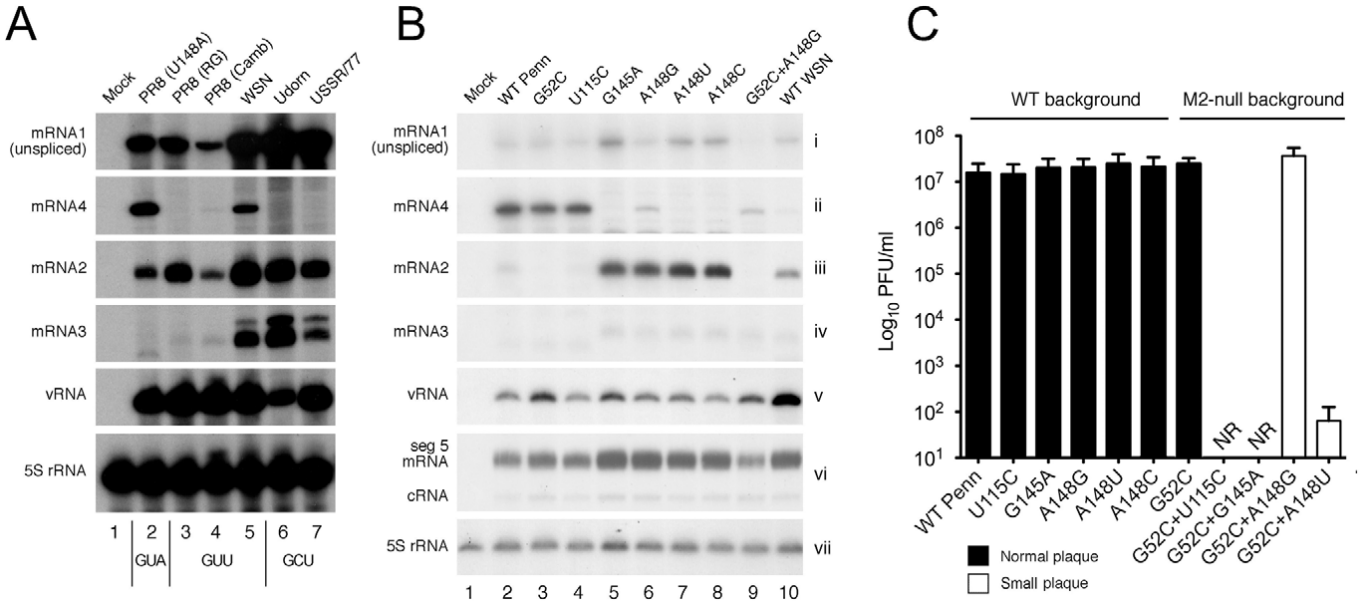
Expression and functional significance of mRNA4 in other strains of IAV. (A, B) Total RNA from cells infected with the indicated viruses at (A) an MOI of 5 and harvested at 6 h p.i. or (B) an MOI of ∼1 and harvested at 9 h p.i. was analysed by RT-primer extension for cellular 5S rRNA and virus-derived RNA species as labeled. (C) Endpoint titres after multicycle replication in MDCK cells of 7+1 reassortant PR8 viruses (PR8 MPenn) with the indicated mutations to Penn segment 7. Values are plotted are the mean + SEM of 3–14 independent rescues. NR; not rescued in 3 attempts. Viruses were also visually classified into normal (black bar) and small (open bar) plaque phenotypes.

The work described above demonstrated that mRNA4 encodes a biologically significant polypeptide that can compensate for loss of M2 expression. The question therefore arose as to whether this might apply to other strains of IAV. The two requisites for M42 expression are production of mRNA4 and the possession of an AUG codon in the appropriate reading frame. mRNA4 was originally discovered in the WSN strain of virus but was not detected in the A/Udorn/72 (Udorn) strain, a difference proposed to result from a single nucleotide difference in the sequences immediately surrounding the splice site: AG/GUU in WSN versus AG/GCU in Udorn ([12]; see Fig. S1, which shows an alignment of the viruses used or discussed in this work, in addition to the consensus sequences of the major virus subtypes that have infected humans this century). To test this prediction, we compared mRNA4 synthesis in a panel of viruses with either GUA, GUU or GCU at the 5′-end of the mRNA4 intron. In agreement with the quality of match with the consensus SD sequence, mRNA4 was not detectable in the two viruses with a GCU sequence: human H3N2 Udorn and H1N1 A/USSR/77 (Fig. 8A, lanes 6 and 7), while it was most abundant in the PR8 U148A mutant (GUA; lane 2). The prediction was also partially supported when mRNA4 synthesis was examined in viruses with a GUU sequence immediately downstream of the SD site. Intermediate quantities of mRNA 4 were detected in RNA from WSN and Cambridge PR8 virus infected cells (lanes 4 and 5; note lower overall amounts of segment 7 mRNAs with the latter virus). Curiously however, mRNA4 was not seen from reverse genetics PR8 (compare lanes 3–5). This was despite segment 7 of this virus only differing from Cambridge PR8 and WSN at two nucleotide positions in the 5′-240 nucleotides, with neither change located near the mRNA4 SD sequence (Fig. S1 and data not shown). There were also notable differences in the amount of mRNA3 produced by the viruses, with WSN and Udorn making abundant quantities, A/USSR/77 rather less and all three PR8 viruses making very little (Fig. 8A). Thus mRNA4 production is predictable by examination of the SD consensus sequence, with GUA>GUU>>GCU, although other unidentified sequence polymorphisms also play a role.

We therefore used this information to interrogate Genbank for IAV segment 7 sequences likely to express M42. As of October 2011, over three-quarters of the 20,236 viruses for which useful segment 7 sequence was available contain the M42 AUG (data not shown). An imperfect initiation context of the M1/M2 AUG codon (which is likely to be necessary to allow leaky ribosomal scanning) is a very highly conserved feature of IAV (only 3 of 17256 sequences covering the M1 AUG have an optimal G at position +4). However, the majority (∼80%) of viruses are unlikely to produce substantial amounts of mRNA4, as they possess an unfavourable AG/GCU or otherwise non-canonical SD sequence. In 1998, Shih and colleagues identified 8 viruses likely to make appreciable amounts of mRNA4 [12]. Now, with an increased number of sequences available as well as a better understanding of the sequence elements necessary for expression of a third biologically active protein from segment 7, we identified around two dozen viruses likely to express M42 (Table 1), by virtue of containing an AUG codon at positions 114–116 and an mRNA4 SD sequence of AG/GU(U/A/G). These mostly fell into three partially overlapping groups: isolates from the early 20^th^ Century (human isolates from the 1930s and two classic fowl plague highly pathogenic avian influenza (HPAI) viruses), viruses that had been adapted to replicate in mice (the WS family, two H3N2 isolates and PR8) and a set of HPAI isolates, mostly from the USA 1983 outbreak [49]. The latter H5N2 grouping seemed the most likely to express large amounts of M42 because of their AG/GUA mRNA4 SD sequence. In addition, the H5N2 outbreak spread widely, persisted for several years and resulted in the culling of 17,000,000 poultry [50], making it an important group of non laboratory-derived viruses, even if represented on Genbank by relatively few sequenced isolates.

**Table 1:** Viruses known or likely to express M42.

Accession	SD	Virus name	Year	Notes
L37797	AG/GUU	A/chicken/Weybridge (H7N7)	1927?	(a)
L25814	AG/GUU	A/NWS/1933 (H1N1)	1933	(b)
M19374	AG/GUU	A/WSN/1933 (H1N1)	1933	(b)
EF467824	AG/GUU	A/Puerto Rico/8/34 (H1N1)	1934	(c)
M55474	AG/GUU	A/chicken/Rostock/1934 (H7N1)	1934	(a)
CY019956	AG/GUU	A/Alaska/1935 (H1N1)	1935	
X08090	AG/GUU	A/Port Chalmers/1/1973-mouse adapted (H3N2)	1973	(b)
CY114430	AG/GUU	A/Bangkok/1/1979 (H3N2)	1979	
CY065977	AG/GUU	A/Philippines/2-MA/1982 (H3N2)	1982	(b)
CY043729	AG/GUU	A/Siena/3/1991 (H3N2)	1991	
FJ784879	AG/GUU	A/chicken/Hunan/1793/2007 (H5N1)	2007	(a)
GU052788	AG/GUA	A/chicken/Pennsylvania/21525/1983 (H5N2)	1983	(a)
FJ610128	AG/GUA	A/chicken/MA/11801/1986 (H5N2)	1986	(a)
CY015074	AG/GUA	A/chicken/Pennsylvania/1/1983 (H5N2)	1983	(a)
GU052756	AG/GUA	A/turkey/Virginia/6962/1983 (H5N2)	1983	(a)
GU052780	AG/GUA	A/chicken/Florida/27716-2/1986 (H5N2)	1986	(a)
FJ610122	AG/GUA	A/chicken/NJ/12508/1986 (H5N2)	1986	(a)
EU980474	AG/GUA	A/chicken/OH/22911-10/1986 (H5N2)	1986	(a)
GU052748	AG/GUA	A/chicken/Pennsylvania/10210/1986 (H5N2)	1986	(a)
FJ357092	AG/GUA	A/guinea fowl/OH/22911-20/1986 (H5N2)	1986	(a)
EU743053	AG/GUA	A/chicken/NY/12004-3/1987 (H5N2)	1987	(a)
EU743234	AG/GUA	A/goose/OH/22911-2/1986 (H5N2)	1986	(a)
GU052772	AG/GUG	A/chicken/Pennsylvania/1370/1983 (H5N2)	1983	(a)
EU743059	AG/GUG	A/chicken/FL/22780-2/1988 (H5N2)	1988	(a)
GU052740	AG/GUG	A/chicken/Pennsylvania/4104/1983 (H5N2)	1983	(a)

20,245 sequences available on the NCBI database were analysed on Oct 10th, 2011, with a repeat search for new sequences of the form AG/GU on July 2^nd^ 2012. The table excludes duplicate sequences, PR8 vaccine reassortants and over 50 cases where supposedly unique sequences showed phylogenetically implausible levels of similarity to PR8 or WSN, suggestive of mistakes in data curation or laboratory contamination of virus stocks.

a Highly pathogenic avian influenza virus, by the molecular definition of possessing a polybasic HA cleavage site.

b Change from AG/GCU SD sequence seen after virus adapted to replicate in mice.

c Mouse adapted virus but original virus sequence not available.

We therefore tested whether the AG/GUA mRNA4 SD consensus of the H5N2 viruses was biologically significant. For biosafety reasons, we used reverse genetics to create a PR8 reassortant (MPenn) with segment 7 from A/chicken/Pennsylvania/10210/1986 (Penn) as well as various mutant derivatives with alterations to the mRNA2 or 4 SD sequences or the M42 AUG codon (Fig. S1), and then analysed their segment 7 mRNA expression profiles. Analysis of viral RNA synthesis showed that, as predicted by the MPenn mRNA4 SD sequence, mRNA4 was the predominant species made from segment 7, accumulating to markedly higher levels than either the unspliced transcript or spliced mRNAs 2 and 3; a reversal of the ratios seen with the ‘prototype’ mRNA 4-expressing virus, WSN, where mRNA4 was the least abundant species (Fig. 8B, compare lanes 2 and 10). Mutations to the mRNA2 and mRNA4 SD sequences had the expected effects. Destruction of the mRNA2 SD sequence by a G52C change reduced mRNA2 accumulation to below detectable levels (lane 3). Removal of the mRNA4 splice site with the G145A change blocked detectable synthesis of mRNA4 with, as before, the side effect of upregulating mRNA2 and mRNA3 production (lane 5). Mutations that weakened the mRNA4 SD consensus (A148G/U or C) dramatically reduced mRNA4 accumulation whilst simultaneously improving synthesis of mRNAs 2 and 3 (lanes 6–9). Also as expected, these changes were specific to segment mRNA, as the levels of segment 7 vRNA and segment 5 mRNA and cRNA were much more consistent between viruses (Fig. 8B).

Next the impact of these changes on virus growth were assessed. The WT MPenn reassortant virus grew well, reaching titres of around 10^7^ PFU/ml (Fig. 8C). Abolition of mRNA2 expression (G52C) had no effect on virus replication; in contrast to the attenuation seen when M2 synthesis was blocked in other virus strains [15], [36]–[39]. Similarly, mutations predicted to block M42 expression by destroying its AUG codon (U115C) or mRNA4 production (G145A) had no effect on virus growth. A similar lack of effect on virus titres were seen from the mutations that attenuated mRNA4 production: A148G, A148U and A148C. However, double mutations targeting both M2 and M42 production were deleterious to virus growth. Viruses lacking the M42 AUG codon or mRNA4 SD sequence could not be rescued (in 3 attempts) in combination with the G52C mRNA 2 SD knockout (Fig. 8C). Moderate downregulation of mRNA4 production by an A148G change in the absence of mRNA 2 synthesis led to a virus that grew to high titres but with a small plaque phenotype, while a more severe downregulation of mRNA4 synthesis with an A148U change resulted in an additional phenotype of very poor growth. Overall, these data indicate that the A/chicken/Pennsylvania/10210/1986 segment 7 expresses two functionally redundant versions of the viral ion channel, either one of which is sufficient to support replication in cultured cells. Thus M42 expression is not peculiar to laboratory adapted viruses but is likely to have been a feature of a major group of HPAI viruses that circulated for four years in North America.

## Discussion

Here, we demonstrate expression of a 14th IAV polypeptide; a variant form of the M2 protein with an alternative ectodomain, encoded by a distinct segment 7 mRNA. This novel protein, M42, can functionally replace M2 and support efficient replication in tissue culture cells and pathogenicity in an animal host, despite showing clear phenotypic differences with respect to its intracellular localization. We have not directly tested M42 for proton conductance but the PR8 MUd virus engineered to express M42 rather than M2 retained amantadine sensitivity (data not shown), providing indirect evidence that the protein retains this function, as expected from its identical transmembrane domain sequence to M2. The ability of M42 to support efficient virus replication despite its inefficient transport to the plasma membrane is interesting in light of current theories regarding the role of M2 in membrane scission [32].

Like the three other IAV “accessory” genes that were discovered long after the virus genome was sequenced (PB1-F2, PB1-N40 and PA-X; [4]–[6]), M42 is clearly non-essential for virus replication, as long as sufficient M2 is expressed. Unlike the additional proteins expressed from the P protein genes, M42 expression is likely to be restricted to a minority of IAV strains under normal conditions, as a result of the suboptimal SD sequence of mRNA4. Examination of the consensus sequences for the major subtypes of IAV that have infected humans in the last century showed that (in consensus, with occasional exceptions) all possess(ed) a weak mRNA4 SD sequence (GCU at the intron boundary) of the type found in Udorn (Fig. S1). However, all these viruses except the current 2009 swine-origin pandemic virus also contain the M42 AUG codon as well as an imperfect Kozak consensus around the M1/M2 AUG codon (Fig. S1), suggesting the potential for M42 expression should mRNA4 expression be present. This perhaps argues that there are environments in which it is advantageous for IAV to shift the balance of segment 7 splicing away from the normal mRNA2/M2 route to increased mRNA4/M42. In this respect it is noteworthy that increased mRNA4 synthesis has been selected for on at least three, probably four, occasions on different virus backgrounds during adaptation to growth in mice (Table 1). Also, given the Golgi-biased localisation of M42, it is tempting to draw a link between the requirement for pH-modulation of the Golgi during intracellular transport of HA molecules with polybasic cleavage sites [25], [51] and the overrerpresentation of HPAI viruses in the list of those likely to express M42. We also speculate that the altered antigenicity of the M42 ectodomain might provide the virus with a route to escape selection pressure imposed by a vaccine directed against the M2e sequence, given that in many viruses, a single nucleotide change would be predicted to alter the balance of splicing towards M42.

The viruses in which we can be reasonably confident of M42 expression represent a very small minority (∼0.2%) of the available sequences. However, there are two further considerations that may render M42 expression more widespread in IAV than our conservative prediction in Table 1. Firstly, we do not yet fully understand what controls segment 7 splicing. A sizeable number of viruses (around 15%; mostly from avian hosts) have an mRNA4 SD sequence of AG/GCA. An A at position+3 clearly promotes more efficient use of the splice site when position +2 is U but it remains to be determined if it is sufficient to override a C at +2. Furthermore, the differences in relative splicing seen between PR8 and WSN viruses make it clear that sequence elements outside of the core consensus splice sites affect their use; these sequences are identical in the two viruses but their splicing patterns are very different. Analysis of a 7+1 PR8:WSN reassortant indicates that the difference is intrinsic to segment 7 (HW, PD, unpublished data) but we have not yet identified the sequence determinants. Secondly, there are many precedents for cell-type dependence of alternative splicing in cellular mRNAs [52] so it may be that in some host species and/or cell types, M42 expression is present in a wider array of IAV strains. Further experiments are required to test these hypotheses.

## Materials and Methods

### Ethics statement

Animal experiments were carried out in strict accordance with the recommendations in the Guide for the Care and Use of Laboratory Animals under the auspices of an NIH Animal Care and Use Committee-approved animal study protocol. The protocol was approved by the NIAID Animal Care and Use Committee (Permit Number LID-6E). All efforts were made to minimize suffering.

### Cells, viruses, plasmids and antisera

Madin-Darby Canine Kidney, 293T human embryonic kidney and A549 human lung adenocarcinoma cells were cultured according to standard procedures [53]. A reverse genetics clone of influenza PR8 and its M2-null derivative, V7-T9 have been previously described [37], [47]. A clone of A/chicken/Pennsylvania/10210/1986 (GU052748) with the UTRs derived from A/chicken/Pennsylvania/1/1983 (CY015074; where a complete segment sequence was available) was synthesized by Genscript and cloned into pDUAL reverse genetics vector [47]. Further mutants were made using the 8 bidirectional promoter plasmid system described in [47], following oligonucleotide-directed mutagenesis to introduce the desired changes, as indicated in Fig. S1. Primer sequences are available upon request. Non-recombinant Cambridge lineage PR8 virus, A/USSR/77 and A/WSN/33 viruses were obtained from the University of Cambridge Division of Virology's collection of viruses. A/Udorn/72 was a gift from Professor Compans [54]. M2 and M42-GFP tagged expression constructs were produced by cloning the coding sequences of the respective proteins into the KpnI/AgeI sites of pEGFP-N1 (Clontech). An M42-mCherry fusion was made by substituting the EGFP open reading frame with mCherry. Purchased monoclonal antisera were against ß-tubulin (clone YL1/2: AbD-Serotec), GM130 (Clone 610822; BD Transduction Laboratory), GFP (clone JL8, Clontech) and anti-influenza M2 (Clone 14C2, Abcam). Further anti-M2 reagents of a goat polyclonal (G74) raised against the whole protein and a mouse polyclonal raised against the M2 ectodomain (M2e) were the generous gifts of Drs. Alan Hay and Xavier Saelens, respectively. Rabbit polyclonal anti-M1 (A2917) and anti-NP (A2915) have been previously described [55], [56]. Affinity purified anti-M42 specific serum was purchased from Genscript. Rabbits were immunized with a peptide corresponding to the N-terminal 16 amino acids of the protein, MSLQGRTPILRPIRNE (where unique sequences compared to M2 are underlined).

### Virus rescue, growth and titration

Recombinant viruses were rescued by 8 plasmid transfection into 293T cells followed by amplification in MDCK cells as previously described [37]. In some cases, stocks were further amplified by growth in day 10–12 embryonated eggs, also as described [37]. Tissue culture cells were infected by allowing virus to adsorb for 30–60 min in serum free medium. For synchronous analyses of viral RNA and protein synthesis, infections were carried out at an MOI of 3–10. For analyses of virus growth, infections were initiated at low multiplicity and cells overlaid with serum free medium supplemented with 1 µg/ml trypsin (Worthington Biochemicals) and 0.14% bovine serum albumin. Serial passages were performed by infecting 3×10^6^ MDCK cells at an MOI of 0.01. At 48 h p.i., the medium was clarified and 10 µl (of 5 ml) used to infect fresh MDCK cells. This procedure was repeated a further five times.

Plaque assays were carried out in MDCK cells using an Avicel overlay followed by staining with toluidine blue [37], [57]. Plaque areas were measured from scanned images using an oval selection marquee in the program Image J [58] and calibrated with respect to the area of a 6-well dish. HA assays were performed using 1% chicken red blood cells in 96 well plates according to standard procedures [37].

### Mouse infection

Infection of C57BL/6J or BALB/c mice (strains 664 and 1026, JAX Mice and Services) was carried out under animal BSL3 conditions at the National Institutes of Health. Groups of five 9–10 week old female mice were infected intranasally with 100 PFU of virus in 50 µl DMEM under oxygenated isoflurane anesthesia. Mice were individually identified and weighed daily; mice losing 25% or more of their initial body weight were euthanised. Three mice on days 2 and 7 and four mice on day 4 postinfection were euthanised and lungs collected for weight-normalized homogenization and MDCK plaque titration.

### RNA and protein analyses

Total cellular RNA was extracted using Trizol (Sigma) and individual RNA species detected using radiolabelled reverse transcriptase primer extension followed by urea-PAGE and autoradiography as previously described [17], [59]. The primer GAAGGCCCTCCTTTCAGTCC, which targeted nucleotides 885–904 in mRNA sense, was used to detect segment 7 mRNA. Quantitation was performed using Fujifilm imaging plates and a Fujifilm FLA-5000 fluorescent image analyser. Data was analysed using AIDA software (Raytest). SDS-PAGE followed by western blotting was performed according to standard procedures. Blots were developed using infrared fluorescent secondary antibodies and imaged using a LiCor Biosciences Odyssey platform. Cells were stained for immunofluorescence after formaldehyde fixation using primary followed by Alexa-fluor conjugated secondary antibodies (Invitrogen) as previously described [60] and imaged on Zeiss LSM510, Leica SPE or TCS-NT confocal microscopes. Live cell imaging was performed in a temperature-controlled hood and CO_2_-independent medium as previously described [53].

## Supporting Information

Figure S1 **Alignment of IAV segment 7 splice site sequences from strains either used or discussed in this study.** Individual cDNA sequences are shown for PR8 (Reverse genetics (RG) clone EF467824), WSN (CY034133), Udorn (324335) and Penn (GU052748) viruses. Partial consensus sequences for the regions of interest from indicated strains of human-infecting viruses (H1N1, H2N2, H3N2 and pdm2009 viruses as well as human-derived H5N1 post 1997) were generated by multiple alignment of publicly available sequences on GenBank in August 2011. AUG codons are highlighted in bold and underlined. Positions mutated in PR8 or Penn segment 7 in this study are highlighted in red and labeled with arrows. Consensus sequences for cellular splice donor (SD), splice acceptor (SA) and Kozak sequences surrounding AUGs 1 and 2 are shown above the sequences in bold (M: A or C; R: A or G; Y: C or T). Sequences shown to be important for binding the cellular splicing factor ASF/SF2 [62] are also shown. Matches to the cellular consensus are shaded in green.
(TIF)

Video S1 **Live cell imaging of M2 and M42.** A549 cells were transfected with plasmids encoding M42-mCh and M2-GFP, and imaged 16 h p.i. Cells were imaged at 37°C in CO_2_-independent medium on a Leica SPE confocal microscope. The video shows a merged image of red and green channels.
(MOV)

Video S2 **Live cell imaging of M2.** As above (Video S1), but showing the green (M2-GFP) channel only.
(MOV)

Video S3 **Live cell imaging of M42.** As above (Video S1), but showing the red (M42-mCh) channel only.
(MOV)

## References

[1] Salomon R, Webster RG (2009) The influenza virus enigma. Cell 136: 402–410. 19203576 10.1016/j.cell.2009.01.029 PMC2971533

[2] Du L, Zhou Y, Jiang S (2010) Research and development of universal influenza vaccines. Microbes Infect 12: 280–286. 20079871 10.1016/j.micinf.2010.01.001

[3] Fiers W, De Filette M, El Bakkouri K, Schepens B, Roose K, et al (2009) M2e-based universal influenza A vaccine. Vaccine 27: 6280–6283. 19840661 10.1016/j.vaccine.2009.07.007

[4] Wise HM, Foeglein A, Sun J, Dalton RM, Patel S, et al (2009) A complicated message: Identification of a novel PB1-related protein translated from influenza A virus segment 2 mRNA. J Virol 83: 8021–8031. 19494001 10.1128/JVI.00826-09 PMC2715786

[5] Jagger BW, Wise HM, Kash JC, Walters KA, Wills NM, et al (2012) An overlapping protein-coding region in influenza A virus segment 3 modulates the host response. Science 337: 199–204. 22745253 10.1126/science.1222213 PMC3552242

[6] Chen W, Calvo PA, Malide D, Gibbs J, Schubert U, et al (2001) A novel influenza A virus mitochondrial protein that induces cell death. Nat Med 7: 1306–1312. 11726970 10.1038/nm1201-1306

[7] Wise HM, Barbezange C, Jagger BW, Dalton RM, Gog JR, et al (2011) Overlapping signals for translational regulation and packaging of influenza A virus segment 2. Nucleic Acids Res 39: 7775–7790. 21693560 10.1093/nar/gkr487 PMC3177217

[8] Inglis SC, Gething MJ, Brown CM (1980) Relationship between the messenger RNAs transcribed from two overlapping genes of influenza virus. Nucleic Acids Res 8: 3575–3589. 7433101 10.1093/nar/8.16.3575 PMC324176

[9] Lamb RA, Lai CJ (1980) Sequence of interrupted and uninterrupted mRNAs and cloned DNA coding for the two overlapping nonstructural proteins of influenza virus. Cell 21: 475–485. 7407920 10.1016/0092-8674(80)90484-5

[10] Inglis SC, Brown CM (1981) Spliced and unspliced RNAs encoded by virion RNA segment 7 of influenza virus. Nucleic Acids Res 9: 2727–2740. 6169001 10.1093/nar/9.12.2727 PMC326888

[11] Lamb RA, Lai CJ, Choppin PW (1981) Sequences of mRNAs derived from genome RNA segment 7 of influenza virus: colinear and interrupted mRNAs code for overlapping proteins. Proc Natl Acad Sci U S A 78: 4170–4174. 6945577 10.1073/pnas.78.7.4170 PMC319750

[12] Shih SR, Suen PC, Chen YS, Chang SC (1998) A novel spliced transcript of influenza A/WSN/33 virus. Virus Genes 17: 179–183. 9857991 10.1023/a:1008024909222

[13] Lamb RA, Choppin PW (1981) Identification of a second protein (M2) encoded by RNA segment 7 of influenza virus. Virology 112: 729–737. 7257188 10.1016/0042-6822(81)90317-2

[14] Shih SR, Nemeroff ME, Krug RM (1995) The choice of alternative 5′ splice sites in influenza virus M1 mRNA is regulated by the viral polymerase complex. Proc Natl Acad Sci U S A 92: 6324–6328. 7541537 10.1073/pnas.92.14.6324 PMC41510

[15] Chiang C, Chen GW, Shih SR (2008) Mutations at alternative 5′ splice sites of M1 mRNA negatively affect influenza A virus viability and growth rate. J Virol 82: 10873–10886. 18768984 10.1128/JVI.00506-08 PMC2573221

[16] Jackson D, Lamb RA (2008) The influenza A virus spliced messenger RNA M mRNA3 is not required for viral replication in tissue culture. J Gen Virol 89: 3097–3101. 19008398 10.1099/vir.0.2008/004739-0 PMC4677827

[17] Robb NC, Fodor E (2011) The accumulation of influenza A virus segment 7 spliced mRNAs is regulated by the NS1 protein. J Gen Virol In press. 10.1099/vir.0.035485-0 21918006

[18] Holsinger LJ, Lamb RA (1991) Influenza virus M2 integral membrane protein is a homotetramer stabilized by formation of disulfide bonds. Virology 183: 32–43. 2053285 10.1016/0042-6822(91)90115-r

[19] Sugrue RJ, Hay AJ (1991) Structural characteristics of the M2 protein of influenza A viruses: evidence that it forms a tetrameric channel. Virology 180: 617–624. 1989386 10.1016/0042-6822(91)90075-M PMC7131614

[20] Schnell JR, Chou JJ (2008) Structure and mechanism of the M2 proton channel of influenza A virus. Nature 451: 591–595. 18235503 10.1038/nature06531 PMC3108054

[21] Pielak RM, Chou JJ (2011) Influenza M2 proton channels. Biochim Biophys Acta 1808: 522–529. 20451491 10.1016/j.bbamem.2010.04.015 PMC3108042

[22] Hay AJ, Wolstenholme AJ, Skehel JJ, Smith MH (1985) The molecular basis of the specific anti-influenza action of amantadine. Embo J 4: 3021–3024. 4065098 10.1002/j.1460-2075.1985.tb04038.x PMC554613

[23] Pinto LH, Holsinger LJ, Lamb RA (1992) Influenza virus M2 protein has ion channel activity. Cell 69: 517–528. 1374685 10.1016/0092-8674(92)90452-i

[24] Bukrinskaya AG, Vorkunova NK, Pushkarskaya NL (1982) Uncoating of a rimantadine-resistant variant of influenza virus in the presence of rimantadine. J Gen Virol 60: 61–66. 7097251 10.1099/0022-1317-60-1-61

[25] Sugrue RJ, Bahadur G, Zambon MC, Hall-Smith M, Douglas AR, et al (1990) Specific structural alteration of the influenza haemagglutinin by amantadine. EMBO J 9: 3469–3476. 2209554 10.1002/j.1460-2075.1990.tb07555.x PMC552095

[26] Chen BJ, Leser GP, Jackson D, Lamb RA (2008) The influenza virus M2 protein cytoplasmic tail interacts with the M1 protein and influences virus assembly at the site of virus budding. J Virol 82: 10059–10070. 18701586 10.1128/JVI.01184-08 PMC2566248

[27] Grantham ML, Stewart SM, Lalime EN, Pekosz A (2010) Tyrosines in the influenza A virus M2 protein cytoplasmic tail are critical for production of infectious virus particles. J Virol 84: 8765–8776. 20573832 10.1128/JVI.00853-10 PMC2918991

[28] Iwatsuki-Horimoto K, Horimoto T, Noda T, Kiso M, Maeda J, et al (2006) The cytoplasmic tail of the influenza A virus M2 protein plays a role in viral assembly. J Virol 80: 5233–5240. 16699003 10.1128/JVI.00049-06 PMC1472145

[29] McCown MF, Pekosz A (2005) The influenza A virus M2 cytoplasmic tail is required for infectious virus production and efficient genome packaging. J Virol 79: 3595–3605. 15731254 10.1128/JVI.79.6.3595-3605.2005 PMC1075690

[30] McCown MF, Pekosz A (2006) Distinct domains of the influenza a virus M2 protein cytoplasmic tail mediate binding to the M1 protein and facilitate infectious virus production. J Virol 80: 8178–8189. 16873274 10.1128/JVI.00627-06 PMC1563831

[31] Rossman JS, Jing X, Leser GP, Balannik V, Pinto LH, et al (2010) Influenza virus m2 ion channel protein is necessary for filamentous virion formation. J Virol 84: 5078–5088. 20219914 10.1128/JVI.00119-10 PMC2863831

[32] Rossman JS, Jing X, Leser GP, Lamb RA (2010) Influenza virus M2 protein mediates ESCRT-independent membrane scission. Cell 142: 902–913. 20850012 10.1016/j.cell.2010.08.029 PMC3059587

[33] Parks GD, Hull JD, Lamb RA (1989) Transposition of domains between the M2 and HN viral membrane proteins results in polypeptides which can adopt more than one membrane orientation. J Cell Biol 109: 2023–2032. 2553741 10.1083/jcb.109.5.2023 PMC2115837

[34] Parks GD, Lamb RA (1991) Topology of eukaryotic type II membrane proteins: importance of N-terminal positively charged residues flanking the hydrophobic domain. Cell 64: 777–787. 1997206 10.1016/0092-8674(91)90507-u

[35] Park EK, Castrucci MR, Portner A, Kawaoka Y (1998) The M2 ectodomain is important for its incorporation into influenza A virions. J Virol 72: 2449–2455. 9499106 10.1128/jvi.72.3.2449-2455.1998 PMC109545

[36] Cheung TK, Guan Y, Ng SS, Chen H, Wong CH, et al (2005) Generation of recombinant influenza A virus without M2 ion-channel protein by introduction of a point mutation at the 5′ end of the viral intron. J Gen Virol 86: 1447–1454. 15831957 10.1099/vir.0.80727-0

[37] Hutchinson EC, Curran MD, Read EK, Gog JR, Digard P (2008) Mutational analysis of cis-acting RNA signals in segment 7 of influenza A virus. J Virol 82: 11869–11879. 18815307 10.1128/JVI.01634-08 PMC2583641

[38] Watanabe T, Watanabe S, Ito H, Kida H, Kawaoka Y (2001) Influenza A virus can undergo multiple cycles of replication without M2 ion channel activity. J Virol 75: 5656–5662. 11356973 10.1128/JVI.75.12.5656-5662.2001 PMC114278

[39] Takeda M, Pekosz A, Shuck K, Pinto LH, Lamb RA (2002) Influenza a virus M2 ion channel activity is essential for efficient replication in tissue culture. J Virol 76: 1391–1399. 11773413 10.1128/JVI.76.3.1391-1399.2002 PMC135863

[40] Wang R, Song A, Levin J, Dennis D, Zhang NJ, et al (2008) Therapeutic potential of a fully human monoclonal antibody against influenza A virus M2 protein. Antiviral Res 80: 168–177. 18598723 10.1016/j.antiviral.2008.06.002

[41] Zebedee SL, Lamb RA (1988) Influenza A virus M2 protein: monoclonal antibody restriction of virus growth and detection of M2 in virions. J Virol 62: 2762–2772. 2455818 10.1128/jvi.62.8.2762-2772.1988 PMC253710

[42] Zhang M, Zharikova D, Mozdzanowska K, Otvos L, Gerhard W (2006) Fine specificity and sequence of antibodies directed against the ectodomain of matrix protein 2 of influenza A virus. Mol Immunol 43: 2195–2206. 16472860 10.1016/j.molimm.2005.12.015

[43] Zhirnov OP, Konakova TE, Wolff T, Klenk HD (2002) NS1 protein of influenza A virus down-regulates apoptosis. J Virol 76: 1617–1625. 11799156 10.1128/JVI.76.4.1617-1625.2002 PMC135891

[44] Hughey PG, Compans RW, Zebedee SL, Lamb RA (1992) Expression of the influenza A virus M2 protein is restricted to apical surfaces of polarized epithelial cells. J Virol 66: 5542–5552. 1501289 10.1128/jvi.66.9.5542-5552.1992 PMC289113

[45] Jackson IJ (1991) A reappraisal of non-consensus mRNA splice sites. Nucleic Acids Res 19: 3795–3798. 1713664 10.1093/nar/19.14.3795 PMC328465

[46] Kozak M (1986) Point mutations define a sequence flanking the AUG initiator codon that modulates translation by eukaryotic ribosomes. Cell 44: 283–292. 3943125 10.1016/0092-8674(86)90762-2

[47] de Wit E, Spronken MI, Bestebroer TM, Rimmelzwaan GF, Osterhaus AD, et al (2004) Efficient generation and growth of influenza virus A/PR/8/34 from eight cDNA fragments. Virus Res 103: 155–161. 15163504 10.1016/j.virusres.2004.02.028

[48] De Filette M, Ysenbaert T, Roose K, Schotsaert M, Roels S, et al (2011) Antiserum against the conserved nine amino acid N-terminal peptide of influenza A virus matrix protein 2 is not immunoprotective. J Gen Virol 92: 301–306. 20965983 10.1099/vir.0.027086-0

[49] Suarez DL, Senne DA (2000) Sequence analysis of related low-pathogenic and highly pathogenic H5N2 avian influenza isolates from United States live bird markets and poultry farms from 1983 to 1989. Avian Dis 44: 356–364. 10879916

[50] Lupiani B, Reddy SM (2009) The history of avian influenza. Comp Immunol Microbiol Infect Dis 32: 311–323. 18533261 10.1016/j.cimid.2008.01.004

[51] Ohuchi M, Cramer A, Vey M, Ohuchi R, Garten W, et al (1994) Rescue of vector-expressed fowl plague virus hemagglutinin in biologically active form by acidotropic agents and coexpressed M2 protein. J Virol 68: 920–926. 8289394 10.1128/jvi.68.2.920-926.1994 PMC236529

[52] Blencowe BJ, Ahmad S, Lee LJ (2009) Current-generation high-throughput sequencing: deepening insights into mammalian transcriptomes. Genes Dev 23: 1379–1386. 19528315 10.1101/gad.1788009

[53] Amorim MJ, Bruce EA, Read EK, Foeglein A, Mahen R, et al (2011) A Rab11- and microtubule-dependent mechanism for cytoplasmic transport of influenza A virus viral RNA. J Virol 85: 4143–4156. 21307188 10.1128/JVI.02606-10 PMC3126276

[54] Roberts PC, Lamb RA, Compans RW (1998) The M1 and M2 proteins of influenza A virus are important determinants in filamentous particle formation. Virology 240: 127–137. 9448697 10.1006/viro.1997.8916

[55] Amorim MJ, Read EK, Dalton RM, Medcalf L, Digard P (2007) Nuclear export of influenza A virus mRNAs requires ongoing RNA polymerase II activity. Traffic 8: 1–11. 17132145 10.1111/j.1600-0854.2006.00507.x

[56] Noton SL, Medcalf E, Fisher D, Mullin AE, Elton D, et al (2007) Identification of the domains of the influenza A virus M1 matrix protein required for NP binding, oligomerization and incorporation into virions. J Gen Virol 88: 2280–2290. 17622633 10.1099/vir.0.82809-0 PMC2884976

[57] Matrosovich M, Matrosovich T, Garten W, Klenk HD (2006) New low-viscosity overlay medium for viral plaque assays. Virol J 3: 63. 16945126 10.1186/1743-422X-3-63 PMC1564390

[58] Abramoff MD, Magelhaes PJ, Ram SJ (1996) Image processing with ImageJ. Biophotonics International 11: 36–42.

[59] Robb NC, Jackson D, Vreede FT, Fodor E (2010) Splicing of influenza A virus NS1 mRNA is independent of the viral NS1 protein. J Gen Virol 91: 2331–2340. 20519456 10.1099/vir.0.022004-0

[60] Elton D, Simpson-Holley M, Archer K, Medcalf L, Hallam R, et al (2001) Interaction of the influenza virus nucleoprotein with the cellular CRM1-mediated nuclear export pathway. J Virol 75: 408–419. 11119609 10.1128/JVI.75.1.408-419.2001 PMC113933

[61] Zebedee SL, Lamb RA (1989) Growth restriction of influenza A virus by M2 protein antibody is genetically linked to the M1 protein. Proc Natl Acad Sci U S A 86: 1061–1065. 2915973 10.1073/pnas.86.3.1061 PMC286621

[62] Shih SR, Krug RM (1996) Novel exploitation of a nuclear function by influenza virus: the cellular SF2/ASF splicing factor controls the amount of the essential viral M2 ion channel protein in infected cells. Embo J 15: 5415–5427. 8895585 PMC452284

