# Supplementary figures and images for: Identification of a Novel Splice Variant Form of the Influenza A Virus M2 Ion Channel with an Antigenically Distinct Ectodomain
Source: PLoS Pathog. 2012 Nov 1;8(11):e1002998. doi: 10.1371/journal.ppat.1002998 (PMC3486900; doi:10.1371/journal.ppat.1002998)

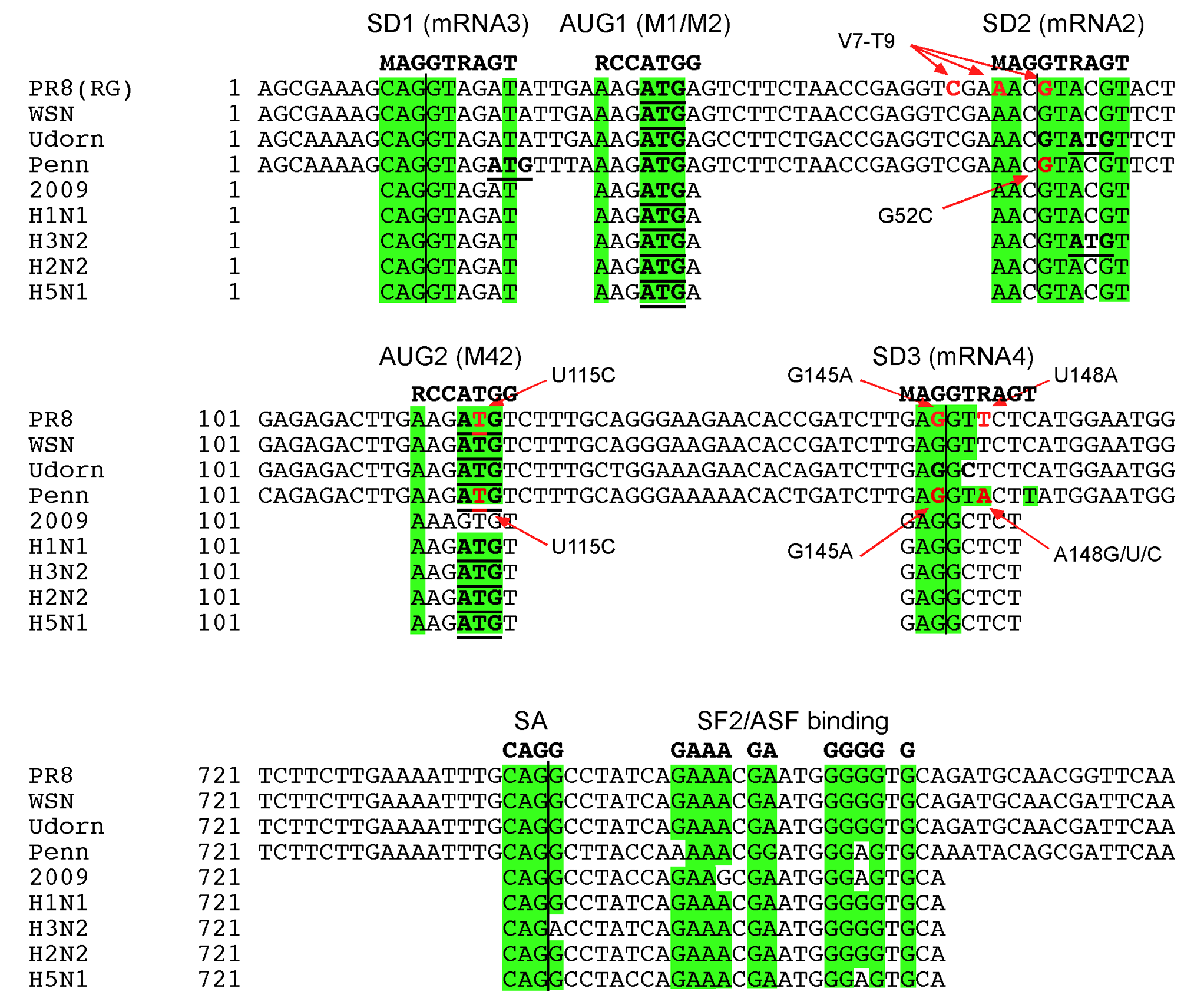

Supplement: Figure S1 — Alignment of IAV segment 7 splice site sequences from strains either used or discussed in this study. Individual cDNA sequences are shown for PR8 (Reverse genetics (RG) clone EF467824), WSN (CY034133), Udorn (324335) and Penn (GU052748) viruses. Partial consensus sequences for the regions of interest from indicated strains of human-infecting viruses (H1N1, H2N2, H3N2 and pdm2009 viruses as well as human-derived H5N1 post 1997) were generated by multiple alignment of publicly available sequences on GenBank in August 2011. AUG codons are highlighted in bold and underlined. Positions mutated in PR8 or Penn segment 7 in this study are highlighted in red and labeled with arrows. Consensus sequences for cellular splice donor (SD), splice acceptor (SA) and Kozak sequences surrounding AUGs 1 and 2 are shown above the sequences in bold (M: A or C; R: A or G; Y: C or T). Sequences shown to be important for binding the cellular splicing factor ASF/SF2 [62] are also shown. Matches to the cellular consensus are shaded in green. (TIF) [file ppat.1002998.s001.tif]
